# Supplementary material for: Predictors of maternal and newborn health service utilization across the continuum of care in Ethiopia: A multilevel analysis
Source: PLoS One. 2022 Feb 25;17(2):e0264612. doi: 10.1371/journal.pone.0264612 (PMC8880850; doi:10.1371/journal.pone.0264612)
Supplement: Attachment — Submitted filename: Review comments for PONE-D-20-37879.docx [file pone.0264612.s003.docx]

Review comments for **PONE-D-20-37879**

Overall:

This is an interesting paper and I believe the findings are important in the context of Ethiopia. However, I do see a lot of areas that need major/significant revision. I also see a lot of grammatical errors throughout the manuscript. Hence, I would suggest that the authors first address the following issues before the manuscript can be considered for publication in PLOS ONE.

Introduction:

- The following sentence needs to be revised in order to clarify what you mean
  - “The continuum of maternal and newborn care provides a framework for delivering high-impact interventions organized in health service packages, ensuring appropriate linkages between family and community care, outreach and outpatient services, and the facility level across the tier to prevent maternal mortality (2).”
- CoC is not defined at first mention in the main body of the text. Please define all acronyms at first mention within the body of the text.
- I do not think that calling individual, interpersonal and household level determinants of service use “demand-side” factors is an appropriate use of the term. Instead of classifying factors as “demand-side” or “supply-side”. I would suggest just calling them what they are (i.e. individual factors, interpersonal factors, household factors, etc.)
- The author mentions that the “evidence on factors associated with adherence to the continuum of maternal and newborn health care is not widely available”. While this is largely true, there are studies done in low- and middle-income countries that looked at determinants of continuum of care. Please cite some of these studies and briefly talk about their findings to inform your study in the introduction. I see that you cite other relevant studies in the discussion section. Also, briefly mention them in the introduction section. Below is a list of references to get started:
  - Determinants of continuum of care for maternal, newborn and child health services in rural Khammouane, Lao PDR by Saki Sakuma, Junko Yasuoko, Khampheng Phongluxa and Masamine Jimba (PLOS ONE; <https://doi.org/10.1371/journal.pone.0215635>)
  - Assessing the Continuum of Care Pathway for Maternal Health in South Asia and Sub-Saharan Africa by Kavita Singh, William T. Story and Allisyn C. Moran (Maternal Child Health Journal; doi: [10.1007/s10995-015-1827-6](https://dx.doi.org/10.1007%2Fs10995-015-1827-6))
  - Factors associated with the continuum of care for maternal, newborn and child health in The Gambia: a cross-sectional study using Demographic and Health Survey 2013 by Jiyoung Oh, Juyoung Moon, Jae Wook Choi and Kyunghee Kim (BMJ Open; <http://dx.doi.org/10.1136/bmjopen-2019-036516>)
  - Analysis of dropout across the continuum of maternal health care in Tanzania: findings from a cross-sectional household survey by Diwakar Mohan, Amnesty E LeFevre, Asha George, Rose Mpembeni, Eva Bazant, Neema Rusibamayila, Japhet Killewo, Peter J Winch and Abdullah H Baqui (Health Policy and Planning; <https://doi.org/10.1093/heapol/czx005>)
  - Levels and determinants of continuum of care for maternal and newborn health in Cambodia-evidence from a population-based survey by Wenjuan Wang and Rathavuth Hong (BMC Pregnancy and Childbirth; doi: [10.1186/s12884-015-0497-0](https://dx.doi.org/10.1186%2Fs12884-015-0497-0))
- The following sentence is incomplete and grammatically incorrect. Please check throughout the manuscript to correct errors like this.
  - “Why the utilization of maternal and newborn health services has not improved in Ethiopia despite the huge national efforts and investments made in the last decade.”

Methods:

- For readers who may not be familiar with “Anderson’s health-seeking behavior model”, please briefly describe the model in the text.
- The author states that the outcome variable of interest was the uptake of the CoC. However, this seems like an oversimplification of what the authors actually did. I see at least three outcome variables: ANC 4+ as the outcome, continuum of care up to delivery and continuum of care through postnatal care. Please describe each outcome variable separately and in detail in the opening paragraph of the “Measurement” sub-section.
- In Table 1, the descriptions for SBA and PNC are ambiguous. For example, defining them as “proportion of women” seems incorrect as you are looking at how an individual woman responded to the corresponding questions.
- In Table 1, the author mentions that PNC included pre-discharge care at the health facility as well as care provided after discharge. Theoretically, determinants for pre-discharge care at the facility and determinants for care after discharge or at home should be treated differently. However, if you decide to keep them together, I would mention how the nature of the determinants could be different for pre-discharge care and after-discharge/home care in the discussion/limitations section.
- I would advise reconsidering the variable “Complete ANC” in the analysis models. This is because the direction of the effect between Complete ANC and ANC4+ is unclear. The authors are trying to predict ANC4+ but ANC4+ may in turn cause Complete ANC. Reverse causality causes the model to be endogenous and estimates unreliable. I would suggest omitting “Complete ANC” entirely when predicting ANC4+.
- Also, have the authors checked for multicollinearity between model family, pregnant women’s conference, family conversation and maternity waiting homes? I wonder if there is high collinearity between these variables and if there are, it could throw off standard errors. If there is high collinearity, I suggest dropping variables that are very highly collinear.
- The three sequential models are fine to use but you could also just use the entire sample for all three models:
  - Model 1:
    - Outcome = 1 if ANC4+
    - Outcome = 0 if not
  - Model 2:
    - Outcome = 1 if ANC4+ & SBA
    - Outcome = 0 if not
  - Model 3:
    - Outcome = 1 if ANC4+ & SBA & PNC
    - Outcome = 0 if not
  - If you decide to keep the three sequential models, make sure to be clear that the findings from Model 2 and Model 3 apply to a sub-sample. This means that based on your findings (from Model 2 and Model 3), program decision-makers can gain insight on a sub-population of women who have followed through to ANC (Model 2) or delivery (Model 3) but not about the general population of pregnant women. However, if you decide to use the alternative models described in the bullet points above, you can have the advantage of generalizing your findings to the general population of pregnant women.

Results:

- There are a few places where the author explains what the findings “mean”. I would suggest reserving interpretations of the findings to the discussion section. The results section should just report the findings and not try to interpret them.
- I don’t think it’s appropriate to say “CoC at the antepartum stage”. Just using antenatal care is not a “continuum of care”.
- Be clear that Model 2 and Model 3 need to be interpreted in the context of the sub-sample. For example, say “In Model 3, administrative region, wealth quintile, being model family, having booked ANC in the first trimester and having received complete ANC remained significantly associated with complete COC at the postnatal stage among women who received four or more ANC visits and skilled birth attendance.”

Discussion:

- Overall, the discussion section is well-organized, rich and clearly written.
- If you decide to keep the three sequential models, I would ask the authors make it very clear in the discussion section that the findings (particularly from Model 2 and Model 3) are not generalizable to the larger population. Rather, those findings (from Model 2 and Model 3) specifically apply to a sub-sample of women who have followed through to ANC (Model 2) or delivery (Model 3). Hence, programmatic implications are only applicable to those women who have followed through to ANC or delivery and not to the general population of pregnant women.
- I would also focus on fixing grammatical errors and awkward sentences.
- Lastly, I would suggest expanding the discussion of methodological limitations a little bit (i.e. potential issues of endogeneity).
