# Supplementary material for: Predictors of maternal and newborn health service utilization across the continuum of care in Ethiopia: A multilevel analysis
Source: PLoS One. 2022 Feb 25;17(2):e0264612. doi: 10.1371/journal.pone.0264612 (PMC8880850; doi:10.1371/journal.pone.0264612)
Supplement: Attachment — Submitted filename: Point-by-point response letter.docx [file pone.0264612.s004.docx]

**A point-by-point response to reviewer/editorial**

**Journal: PLOS ONE**

PONE-D-20-37879
Predictors of maternal and newborn health service utilization across the continuum of care in Ethiopia: a multilevel analysis
PLOS ONE

*The authors would like to appreciate and thank the reviewers and editors for the constructive comments. Our point-by-point responses are below each of the comments in italics.*

**Journal Requirements:**

When submitting your revision, we need you to address these additional requirements.

1. Please ensure that your manuscript meets PLOS ONE's style requirements, including those for file naming. The PLOS ONE style templates can be found at

https://journals.plos.org/plosone/s/file?id=wjVg/PLOSOne_formatting_sample_main_body.pdf and <https://journals.plos.org/plosone/s/file?id=ba62/PLOSOne_formatting_sample_title_authors_affiliations.pdf>

*We make sure that this version of the manuscript conforms to the journal style*

2. We note that your study is closely related to the following publication, on which you are an author:

<https://bmcpregnancychildbirth.biomedcentral.com/articles/10.1186/s12884-020-03254-7>

Although you have briefly cited the above study in the discussion section of your article, we feel that the scientific rationale of the current study and the contribution that it makes to the field should be better justified.  Therefore, please cite and discuss the above study in the introduction and discussion sections of your manuscript, clarifying how the present work is related to the previously published paper.

Please note that our second publication criterion states that "If a submitted study replicates or is very similar to previous work, authors must provide a sound scientific rationale for the submitted work and clearly reference and discuss the existing literature. Submissions that replicate or are derivative of existing work will likely be rejected if authors do not provide adequate justification.  http://www.plosone.org/static/publication.action#results.

Thank you for your attention to this request.

*Thanks a lot for raising your concerns. The previous publication examined predictors of postpartum use. However, this manuscript examines predictors of the continuum of maternal and newborn care across the antepartum, intrapartum, and postpartum stages. Though both studies used the same data sources, they examined different objectives and presented new knowledge to audiences. Accordingly, all figures included in this manuscript are original and have not been published in previous publications. In the revised version of this manuscript, we referenced and discussed the previous publication and avoid textual overlaps.*

3. Your ethics statement should only appear in the Methods section of your manuscript. If your ethics statement is written in any section besides the Methods, please move it to the Methods section and delete it from any other section. Please ensure that your ethics statement is included in your manuscript, as the ethics statement entered into the online submission form will not be published alongside your manuscript.

*Comment is well taken and the ethics statement is moved to the Methods section.*

4. We noticed you have some minor occurrence of overlapping text with the following previous publication(s), which needs to be addressed:

- <https://bmcpregnancychildbirth.biomedcentral.com/articles/10.1186/s12884-020-03254-7>

The text that needs to be addressed involves the results section.

In your revision ensure you cite all your sources (including your own works), and quote or rephrase any duplicated text outside the methods section. Further consideration is dependent on these concerns being addressed.

*Thanks a lot for your valuable comments. In the revised version of this manuscript, to avoid textual overlaps regarding socio-demographic characteristics we dropped this section and referenced and discussed the previous publication.*

**Reviewers' comments:

Reviewer #1:** 
**Overall:**
This is an interesting paper and I believe the findings are important in the context of Ethiopia. However, I do see a lot of areas that need major/significant revision. I also see a lot of grammatical errors throughout the manuscript. Hence, I would suggest that the authors first address the following issues before the manuscript can be considered for publication in PLOS ONE.

**Introduction:**
• The following sentence needs to be revised in order to clarify what you mean
o “The continuum of maternal and newborn care provides a framework for delivering high-impact interventions organized in health service packages, ensuring appropriate linkages between family and community care, outreach and outpatient services, and the facility level across the tier to prevent maternal mortality (2).”

*Thanks for the comment. To clarify for the readers, it is revised as follows, “The continuum of maternal and newborn care is highlighted as a framework for delivering high-impact interventions across the pregnancy, childbirth, and postpartum life-course and the level of service delivery to prevent maternal and newborn mortalities (2).”*

• CoC is not defined at first mention in the main body of the text. Please define all acronyms at first mention within the body of the text.

*Comment well taken and addressed in this version.*

• I do not think that calling individual, interpersonal and household level determinants of service use “demand-side” factors is an appropriate use of the term. Instead of classifying factors as “demand-side” or “supply-side”. I would suggest just calling them what they are (i.e. individual factors, interpersonal factors, household factors, etc.)

*Many thanks for the comment. Comment well taken and addressed in this version.*

• The author mentions that the “evidence on factors associated with adherence to the continuum of maternal and newborn health care is not widely available”. While this is largely true, there are studies done in low- and middle-income countries that looked at determinants of continuum of care. Please cite some of these studies and briefly talk about their findings to inform your study in the introduction. I see that you cite other relevant studies in the discussion section. Also, briefly mention them in the introduction section. Below is a list of references to get started:
o Determinants of continuum of care for maternal, newborn and child health services in rural Khammouane, Lao PDR by Saki Sakuma, Junko Yasuoko, Khampheng Phongluxa and Masamine Jimba (PLOS ONE; https://doi.org/10.1371/journal.pone.0215635)
o Assessing the Continuum of Care Pathway for Maternal Health in South Asia and Sub-Saharan Africa by Kavita Singh, William T. Story and Allisyn C. Moran (Maternal Child Health Journal; doi: 10.1007/s10995-015-1827-6)
o Factors associated with the continuum of care for maternal, newborn and child health in The Gambia: a cross-sectional study using Demographic and Health Survey 2013 by Jiyoung Oh, Juyoung Moon, Jae Wook Choi and Kyunghee Kim (BMJ Open; http://dx.doi.org/10.1136/bmjopen-2019-036516)
o Analysis of dropout across the continuum of maternal health care in Tanzania: findings from a cross-sectional household survey by Diwakar Mohan, Amnesty E LeFevre, Asha George, Rose Mpembeni, Eva Bazant, Neema Rusibamayila, Japhet Killewo, Peter J Winch and Abdullah H Baqui (Health Policy and Planning; https://doi.org/10.1093/heapol/czx005)
o Levels and determinants of continuum of care for maternal and newborn health in Cambodia-evidence from a population-based survey by Wenjuan Wang and Rathavuth Hong (BMC Pregnancy and Childbirth; doi: 10.1186/s12884-015-0497-0)

*Many thanks for the valid comments. All these articles are cited in the Background and Discussion sections in the revised version.*

• The following sentence is incomplete and grammatically incorrect. Please check throughout the manuscript to correct errors like this.
o “Why the utilization of maternal and newborn health services has not improved in Ethiopia despite the huge national efforts and investments made in the last decade.”

*Comment well taken and addressed.*

**Methods:**
• For readers who may not be familiar with “Anderson’s health-seeking behavior model”, please briefly describe the model in the text.

*Comment is well taken and a brief description of the model included.*

• The author states that the outcome variable of interest was the uptake of the CoC. However, this seems like an oversimplification of what the authors actually did. I see at least three outcome variables: ANC 4+ as the outcome, continuum of care up to delivery and continuum of care through postnatal care. Please describe each outcome variable separately and in detail in the opening paragraph of the “Measurement” sub-section.

*Comment well taken and addressed as follows, “The outcome variables of interest of the study were the uptake of the CoC at antepartum, intrapartum, and postpartum stages: 1) continuum of care at the antepartum stage is women who received ANC 4+ visits, 2) continuum of care at the intrapartum stage is those women who continued use of skilled birth attendance after receiving ANC4+ visits, and 3) continuum of care at the postpartum stage or complete continuum is those women who received PNC for the mothers and their newborns, within six weeks of their delivery (either in a facility or at home) after receiving both ANC4+ visits and delivered by a skilled assistance.”*

• In Table 1, the descriptions for SBA and PNC are ambiguous. For example, defining them as “proportion of women” seems incorrect as you are looking at how an individual woman responded to the corresponding questions.

*Comment is well taken and addressed in this version.*

• In Table 1, the author mentions that PNC included pre-discharge care at the health facility as well as care provided after discharge. Theoretically, determinants for pre-discharge care at the facility and determinants for care after discharge or at home should be treated differently. However, if you decide to keep them together, I would mention how the nature of the determinants could be different for pre-discharge care and after-discharge/home care in the discussion/limitations section.

*Thanks for the valuable comment. In the discussion section, we included the following sentence as a limitation of this paper. “In defining PNC, we included both postpartum pre-discharge care at the health facility as well as postpartum care provided after discharge to see the full spectrum of uptake of postpartum care. Accordingly, we examined predictors for pre-discharge and after-discharge care together which theoretically be different for pre-discharge care at the facility and care after discharge or at home.”*

• I would advise reconsidering the variable “Complete ANC” in the analysis models. This is because the direction of the effect between Complete ANC and ANC4+ is unclear. The authors are trying to predict ANC4+ but ANC4+ may in turn cause Complete ANC. Reverse causality causes the model to be endogenous and estimates unreliable. I would suggest omitting “Complete ANC” entirely when predicting ANC4+.

*Thanks for the valid and educational comment. It is well taken and Complete ANC is omitted from Model I. The results including the result Table are edited accordingly.*

• Also, have the authors checked for multicollinearity between model family, pregnant women’s conference, family conversation and maternity waiting homes? I wonder if there is high collinearity between these variables and if there are, it could throw off standard errors. If there is high collinearity, I suggest dropping variables that are very highly collinear.

*Thanks a lot for the valid comments. We have checked for multi-collinearity to see the assumption of independence of variables in the multiple regression model and we found out a mean-variance inflation factor (vif) of 1.05 that there is no multi-collinearity problem.*

• The three sequential models are fine to use but you could also just use the entire sample for all three models:
o Model 1:
♣ Outcome = 1 if ANC4+
♣ Outcome = 0 if not
o Model 2:
♣ Outcome = 1 if ANC4+ & SBA
♣ Outcome = 0 if not
o Model 3:
♣ Outcome = 1 if ANC4+ & SBA & PNC
♣ Outcome = 0 if not
o If you decide to keep the three sequential models, make sure to be clear that the findings from Model 2 and Model 3 apply to a sub-sample. This means that based on your findings (from Model 2 and Model 3), program decision-makers can gain insight on a sub-population of women who have followed through to ANC (Model 2) or delivery (Model 3) but not about the general population of pregnant women. However, if you decide to use the alternative models described in the bullet points above, you can have the advantage of generalizing your findings to the general population of pregnant women.

*Thanks for the detailed explanation. We defined the three models exactly how you described them above. Few differences in the sample size for the three models are due to missing values where we recoded “Don’t know” values as missing. In the analysis section, we clearly defined how the outcome variables are coded 1 or 0 for further clarification.*

**Results:**
• There are a few places where the author explains what the findings “mean”. I would suggest reserving interpretations of the findings to the discussion section. The results section should just report the findings and not try to interpret them.

*Comment is well taken and revisited in this version.*

• I don’t think it’s appropriate to say “CoC at the antepartum stage”. Just using antenatal care is not a “continuum of care”.

*Comment is well taken and revisited in this version.*

• Be clear that Model 2 and Model 3 need to be interpreted in the context of the sub-sample. For example, say “In Model 3, administrative region, wealth quintile, being model family, having booked ANC in the first trimester and having received complete ANC remained significantly associated with complete COC at the postnatal stage among women who received four or more ANC visits and skilled birth attendance.”

*Comment well taken and revisited in this version.*
 **Discussion:**
• Overall, the discussion section is well-organized, rich and clearly written.

*Thanks much.*

• If you decide to keep the three sequential models, I would ask the authors make it very clear in the discussion section that the findings (particularly from Model 2 and Model 3) are not generalizable to the larger population. Rather, those findings (from Model 2 and Model 3) specifically apply to a sub-sample of women who have followed through to ANC (Model 2) or delivery (Model 3). Hence, programmatic implications are only applicable to those women who have followed through to ANC or delivery and not to the general population of pregnant women.

*Thanks for the comment. As we explained earlier we defined the three models exactly how you described them above. In this version, we clearly defined how the outcome variables are coded 1 or 0.*

• I would also focus on fixing grammatical errors and awkward sentences.

*Comment well taken and addressed.*

• Lastly, I would suggest expanding the discussion of methodological limitations a little bit (i.e. potential issues of endogeneity).

*Thanks for the valid comment. The following sentences are included in the limitation section of the revised manuscript. “Another limitation of this study would be the presence of unmeasured confounders that would be correlated both with the outcome and predictor variables included in the model, particularly level-2 endogeneity arising from correlations between included individual characteristics and omitted community-level variables and/ or reverse causality of variables that would cause the model to be endogenous [52]. To minimize the potential endogeneity problems, we excluded predictor variables with the unclear direction of the effect with the outcome variables, for instance, “Complete ANC” was excluded from Model I of predicting ANC4+ uptake.” Line 401-8; page 27*

**Reviewer #2:** the authors investigated the predictors of maternal and newborn health service utilization across the continuum of care in Ethiopia. the authors did a great work and have added to the body of knowledge on maternal health.

*Thanks!!*

the authors should edit their writeup and make sure where ever they use an abbreviation for the first time, they first indicate it in full and the abbreviation in bracket.

*Comment is well taken and addressed in this version.*
