# Supplementary material for: Predictors of maternal and newborn health service utilization across the continuum of care in Ethiopia: A multilevel analysis
Source: PLoS One. 2022 Feb 25;17(2):e0264612. doi: 10.1371/journal.pone.0264612 (PMC8880850; doi:10.1371/journal.pone.0264612)
Supplement: Attachment — Submitted filename: Response to Reviewers.docx [file pone.0264612.s005.docx]

A point-by-point response to reviewer/editorial

Journal: PLOS ONE

PONE-D-20-37879R1

Predictors of maternal and newborn health service utilization across the continuum of care in Ethiopia: a multilevel analysis

*The authors would like to appreciate and thank the reviewers and editors for the constructive comments. Our point-by-point responses are below each of the comments in italics.*

Journal Requirements:

Please review your reference list to ensure that it is complete and correct. If you have cited papers that have been retracted, please include the rationale for doing so in the manuscript text, or remove these references and replace them with relevant current references. Any changes to the reference list should be mentioned in the rebuttal letter that accompanies your revised manuscript. If you need to cite a retracted article, indicate the article’s retracted status in the References list and also include a citation and full reference for the retraction notice.

*Thank you for the valid comments. References cited are now edited. Articles under review*

Additional Editor Comments (if provided):

This is an interesting work. They choose 3 indicators and analyse them as a cascade of a continuum of care (CoC) antepartum (4 antenatal visits), intrapartum (skilled birth attendance conditional the woman had the 4 antenatal visits), and postpartum (postnatal care among those who completed the intrapartum step as defined here). Then they present prevalence of these indicators per characteristics and elements chosen from Anderson’s health-seeking behaviour model.

*Thank you so much.*

However, few issues:
1. Please add some description of the administrative division of Ethiopia. At least the reader will understand what is a woreda.

*Comment well taken. A paragraph is added under the Methods section to describe the administrative division of the country. (Line 79-81, page 5)*

2. Please provide 95% confidence intervals (CI) for each step. That could be accomplished with a bar plot for the antepartum, intrapartum and postpartum. Please, make sure that those CI are adjusted for the clustering.

*Comment well taken. The continuum of care coverage across antepartum, intrapartum and postpartum with error bars is presented in Figure 1, separately attached.*

a. Tables 3 and 4 could have those 95% confidence intervals rather than the p-values. Again, make sure those CI are adjusted for the cluster sampling.

*Thank you for the valid comments. The 95% CI* *adjusted for the cluster sampling is included in both Tables 3 and 4.*

3. Table 3 - for education, I believe complete continuum figures are a repetition of the “continued at antenatal care”.

*Thanks a lot for comment. You are right. It was an error. It is now fixed.*

4. Table 5 - Please add a row with the number of observations included in each model

*Thanks. Number of observations in each model is included in Table 5.*

**Comments to the Author**

Reviewer #3: As stated by a previous reviewer, the entire manuscript needs copy editing. Specific issues include:

*Comment well taken and copy edited.*


Line 47-48: This first sentence is confusing and frankly, not necessary.

*Comment well taken and edited accordingly.*


Line 50-51: I believe a modifier may be missing here, such as “level of service delivery needed to prevent…”

*Comment well taken and edited accordingly.*

Line 63: What are “supply-side” factors? Number of physicians/nurses/facilities?

*Comment well taken edited accordingly.*

Line 68: Needs a reference

*Comment well taken.*

Line 124-128: The direction of “no difficulty of distance and transportation arrangement to access medical care” as an “exposure” variable does not make sense given the direction of the other variables; please explain. Additionally, II am not sure that “exposure” is the correct term. Predictor is more appropriate. You were not “exposing” women to these factors with an intervention.

*Thanks for the comment. We checked the adequacy of our sample size using a double population, using case and control or exposure and non-exposed groups to determine the predictor variables of the study.*

Line 134-136: At line 135, “four or more antental care (ANC4+)” appears in item 2 but should appear in item 1, as that is its first use.

*Comment well taken and addressed.*


Line 137: What is PNC? (Spell it out before abbreviating it.)

*Comment well taken and spelt out.*

Line 149: I am surprised you did not include the infant’s birth weight, estimated gestational age at birth, health at birth (did the infant need to stay in a neonatal special care unit, did the infant have a congenital anomaly), or survival. These factors may influence receipt of care at the delivery and postnatal stages. Were these items asked?

*Thanks for the valid comments. Variable infant’s birth weight was collected and included in the analysis; but not significantly associated with COC at postpartum stage. And this is indicated in the revised version of the manuscript. However, we did not collect variables like estimated gestational age at birth, health at birth and/or presence of a congenital anomaly.*

Table 3, 4: What are these proportions? Neither columns nor rows sum to 100% in any group. Also, what is the denominator for the N’s?

*Thank you for the comment. The denominator for Tables 3 and 4 are presented as “****Total’’*** *column at the end of each table. The proportions presented in these tables are actual percentages of each category; their complements are not presented which we believe readers can get by subtracting from 100%.*
